# Failed Dental Implant: When Titanium Fractures

**DOI:** 10.3390/diagnostics13122123

**Published:** 2023-06-20

**Authors:** João Paulo Mendes Tribst, Arie Werner, Erik J. Blom

**Affiliations:** 1 Department of Reconstructive Oral Care, Academic Centre for Dentistry Amsterdam (ACTA), Universiteit van Amsterdam and Vrije Universiteit, 1081 LA Amsterdam, The Netherlands; e.blom@acta.nl; 2 Department of Dental Materials Science, Academic Centre for Dentistry Amsterdam (ACTA), University of Amsterdam and Vrije University Amsterdam, 1081 LA Amsterdam, The Netherlands; a.werner@acta.nl

**Keywords:** dental implants, dental restoration failure, biomaterial, fracture strength, single-tooth implants

## Abstract

Despite the widespread use of titanium implants in orthopedic and dental surgeries, concerns have recently emerged regarding potential deformations and fractures after osseointegration. In a recent clinical case, a titanium implant fractured after successful osseointegration. This fracture occurred despite the absence of any significant trauma or excessive external force applied to the area. The fracture was attributed to a combination of factors, including abutment design flaws, material fatigue, and biomechanical stress imposed on the implant during functional loading. This raises concerns about the long-term durability and reliability of titanium implants, particularly in high-stress areas such as the posterior region or weight-bearing bones. An image was made with scanning electron microscopy showing the fracture region near the prosthetic platform and highlighting the knowledge that despite their ductility, titanium implants can fracture.

Dental implants have emerged as a highly successful and widely used treatment option for replacing missing teeth [1,2]. With their ability to restore oral function and aesthetics, dental implants have revolutionized the field of dentistry [1,2]. However, despite their overall success, failures and complications associated with dental implants can occur, with fractures and mechanical failures being significant concerns. Dental implant failures can be classified into two categories: biological and mechanical. Biological failures primarily involve issues related to osseointegration, peri-implantitis (inflammation and bone loss around the implant), and soft tissue complications. On the other hand, mechanical failures encompass fractures and other structural problems directly associated with the implant components [3,4,5,6].

Among the various types of dental implant failures, fractures and mechanical failures pose particular challenges due to their potential impact on implant stability, function, and patient satisfaction [2,3]. Implant fractures can occur in different parts of the implant system, including the implant body, abutment, or prosthetic components such as the crown or bridge [4,5,6,7]. Mechanical failures in dental implants can result from a combination of factors, including material fatigue [5], design flaws [3], improper implant placement, occlusal overload (excessive force during biting and chewing), or traumatic incidents. These failures can lead to compromised aesthetics, functional limitations, and patient discomfort [2,3].

The present image was produced after the explantation of a clinical case, from a patient who experienced a titanium implant fracture after successful osseointegration. According to the patient report, this fracture occurred despite the absence of any significant trauma or external force applied to the area. The implant fixture dimension was ⌀ 5.4 mm and 9 mm height with a morse-taper connection (Astra Tech AB, Mölndal, Sweden) placed in native bone tissue. The prosthetic design was a straight abutment for a screw-retained individualized abutment made of titanium–aluminum–vanadium. The implant placement was performed in the posterior region, rehabilitating the screw-retained single-crown of the left maxillary second molar. The antagonist was natural dentition with adequate occlusion. The crown was in function for six years since its placement and the fracture became noticeable due to its exacerbated movement.

To evaluate the failed implant, the collected specimen was observed under scanning electron microscopy (XL20; Philips, Eindhoven, The Netherlands) at 40×, 100×, and 500× magnification (Figure 1). This visual analysis was performed to identify the fracture origin and crack pattern. The fracture was attributed to a combination of factors, including abutment design misfit, material fatigue, and biomechanical stress imposed on the implant during functional loading [4,5]. Fractures and mechanical failures not only require additional treatment interventions but result in implant removal and replacement, leading to increased treatment costs, extended treatment duration, and patient dissatisfaction. Therefore, understanding the causes, risk factors, and preventive strategies for implant fractures and mechanical failures is crucial for ensuring long-term implant success [8,9,10].

According to the literature, the fracture of a titanium dental implant is an infrequent complication affecting 0.2% of every 1000 implants [11], and the complete removal of an implant fragment is the most commonly indicated treatment option [11]. In summary, the fracture of an osseointegrated implant is a late complication which can be due to multifactorial etiology, named as a rare but not exceptional problem [6]. In addition, it is worth noting that the majority of images depicting fractured dental implants often consist of X-ray examinations from patients, which inherently lack a three-dimensional inspection capability [2,3,11].

Similar to the presented image, previous reports performed SEM on failed dental implants [7,8]. However, Singh et al. [7] used 3000× magnification, showing only the intergranular fracture, reporting that a large dimple at the center of the implant surface was found to consist of various wavy lines or striations [7], while Shibli et al. [8] investigated a failed implant without fracture features.

Several reasons can increase the incidence and causes of implant fractures [12]. According to a literature review, they can be summarized as poor implant planning, implant–abutment misfit, and overloading. However, implant and prosthetic design were possible contributing factors, while the risk of implant fracture increased over time due fatigue. The authors suggested that tridimensional implant position, diameter and number of implants, inclination, abutment selection, as well as occlusion management are mandatory to ensure long-term survival and success of an implant-supported restoration [12]. Based on that, it is possible to suggest that the abutment misfit in this case should have been improved. This recommendation is supported by the fact that the implant diameter was appropriate and satisfactory, the inclination was suitable for using a straight abutment, and the occlusion was assessed. In this context, the stress concentrated at the connection region exceeded the material’s yield strength, resulting in plastic deformation and subsequent failure of the dental treatment. Therefore, the present image complements the literature, showing a longitudinal crack pattern starting at the platform level and descending in the direction of the implant apex. Further reports should be performed to elucidate the major causes as well as the risk factors associated with titanium fracture mechanics.

## Figures and Tables

**Figure 1:**
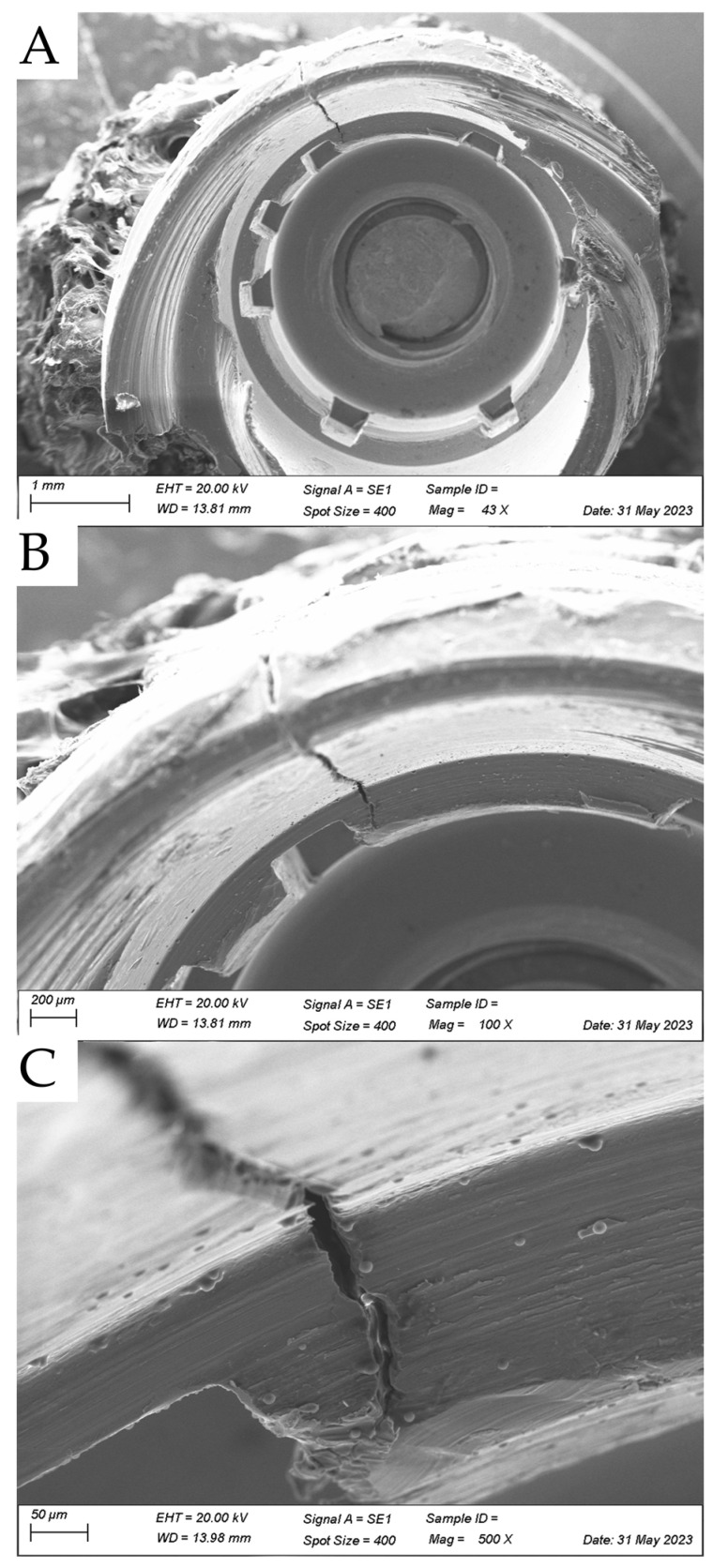
Scanning electron micrograph (SEM) of fractured retrieved dental implant showing a longitudinal crack originated from prosthetic platform at cervical level, descending to the implant apex at (**A**) 40× magnification, (**B**) 100×, and (**C**) 500×.

## Data Availability

All relevant data are within the manuscript.
